# Evaluation of a new point-of-care diagnostic test measuring inflammation in emergency settings

**DOI:** 10.1038/s41598-023-46347-x

**Published:** 2023-11-09

**Authors:** Olivier L. Charansonney, Ghanima Al-Dandachi, Patrick Plaisance, Eric Vicaut

**Affiliations:** 1 Cardiology Department, Centre Hospitalier Sud-Francilien (CHSF), Corbeil-Essonnes, France; 2 Clinical Physiology Department, Hôpital Lariboisière, Paris, France; 3 Emergency Department, Hôpital Lariboisière, Paris, France; 4 Clinical Research Unit, Hôpital Fernand-Widal, Paris, France

**Keywords:** Diagnostic markers, Diagnosis, Diseases

## Abstract

Erythrocyte aggregation kinetics is accelerated in diseases with a strong inflammation component. This study aimed to evaluate whether, in an emergency setting, a new point-of-care test measuring erythrocyte aggregation kinetics (EAK) can identify patients with underlying inflammation. Patients visiting an emergency department and needing a blood exam were successively included. EAK was measured at the point-of-care in 20 s directly on the blood samples collected in regular tubes without any manipulation. The primary measure was EAK’s half-life during the first 5 s (EAK5s). Each patient’s inflammation status was assessed blind to the EAK test results. Receiver Operating Characteristic (ROC) curves for inflammation status were built. 268 patients had their EAK5s measured, and a clear inflammation status was determined for 214 patients (65 had inflammation). Mean EAK5s were 2.18 s and 1.75 s for no inflammation and inflammation groups respectively (p < 0.001). EAK5s appears to be a better inflammation marker than C-Reactive protein (CRP), with an area under the ROC curve of 0.845 compared to 0.806 for CRP (p < 0.0001). The Youden threshold for prediction of inflammation was 1.86 s with 84.6% (78.5–89.9%) specificity and 70.8% (60–81.5%) sensitivity. Point-of-care EAK is an easily measured, immediately available marker of inflammation with a better predictive power than CRP’s.

## Introduction

Medical diagnosis is a complex process: disease specific gold standard tests are not always available and can not be used for every patient, patients often have several diseases, and, especially in emergency settings, medical decisions often need to be made with limited information^1^. Therefore, the diagnostic process requires several steps from the initial clinical evaluation to elaborate and expensive tests (e.g., Magnetic Resonance Imaging). Each diagnostic test with a binary answer (yes/no) is characterized by both sensitivity (the ability to detect a given condition when present) and specificity (the ability to correctly identify the absence of a given condition). Point-of-care diagnostic tests are developed to improve both the first diagnostic orientation and the sensitivity and specificity of further diagnostic tests.

Inflammation is a key component of many disease processes, either during their acute phase (bacterial, viral or parasitic infections, or acute thromboinflammatory diseases) or in their more chronic development (cancer, diabetes…). Therefore, measuring inflammation and its level at the point of care is of great interest for the initial diagnostic orientation. C-Reactive protein (CRP) can be measured at the point of care and can help determine, for instance, the prescription of antibiotics^2,3^. Erythrocyte aggregation is a physiological phenomenon known to be accelerated by inflammation^4,5^. We evaluated the power of a point-of-care measure of erythrocyte aggregation kinetics (EAK) to predict inflammation and compared it to the predictive power of CRP in patients visiting an emergency department (ED).

## Results

### Patient population

Two hundred and seventy-five patients visiting Lariboisière Hospital’s ED representing 57% of the eligible patients were included (11 dayshifts between January and June 2016). Main reasons for non-inclusion were:

- Patients visiting the ED at night,
- Patients refusing to participate,
- Failure to propose to patients that they participate in the study when the ED was overcrowded.

In six patients EAK measures were defective for technical reasons. Two hundred and sixty-nine patients (mean/median age 56.3/58 years, 53.5% women) were finally included.

The main reasons for visiting the ED were abdominal pain (18.2%), chest pain (14.9%), headache/neurological condition (14.5%), dyspnea (13.8%), and trauma/accident/poison (13.8%), with 12% having two or three reasons. One hundred and twenty-five patients (47%) were hospitalized after their ED visit. One patient died during the follow-up period.

### EAK and inflammation

A clear final inflammation status was determined for 214 patients (80%), with 65 patients (30.4%) assessed as having inflammation. In 50.8% of these patients, inflammation was associated with an infection (bacterial, viral, or parasitic), in 13.9% with a digestive tract inflammation and in 10.8% with an active cancer.

The 5s model explained at least 99% of the variance (r^2^ ≥ 0.99) in 90.7% of the patients (and at least 94% of the variance in the entire study population). The 1.5s model explained at least 99% for the remaining patients.

Mean EAK5s values were 2.18 ± 0.39 s and 1.75 ± 0.29 s for no inflammation and inflammation groups respectively (p < 0.001).

The ROC curve showed that EAK5s’s predictive power for inflammation is good with an area under the curve of 0.83 (95% CI: 0.77–0.89).

The Youden threshold was 1.857 s with 84.6% (78.5–89.9%) specificity and 70.8% (60—81.5%) sensitivity.

Using this threshold, we saw that the positive predictive value (ppv) of EAK5s was 66.7% and the negative predictive value was 86.9%. *In other words, an EAK5s greater than 1.857 s excluded inflammation with a probability of 86.9%.*

**Figure 1:**
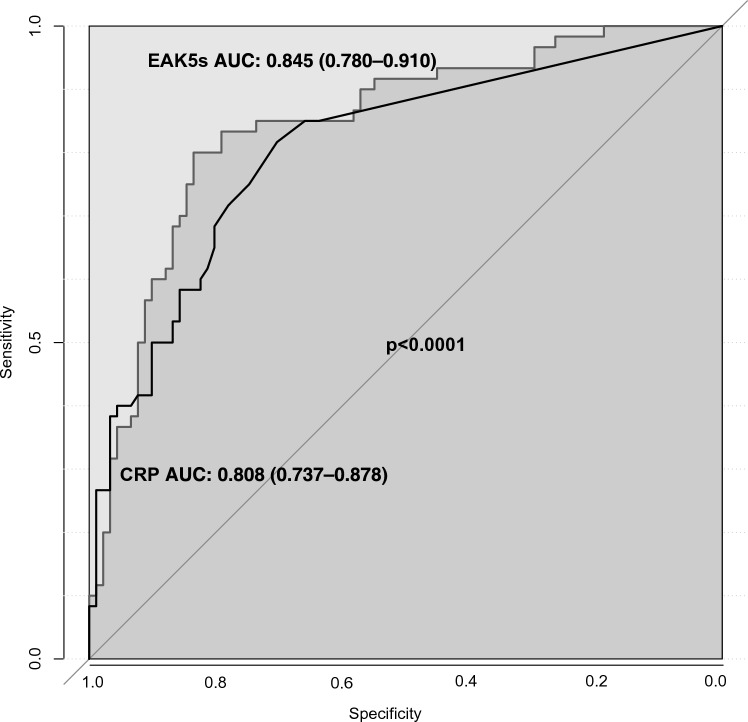
comparison of CRP (black curve) and EAK5s (grey curve). Delong's test of two correlated curves p < 0.0001. *EAK5s* EAK derived index at 5 s, *AUC* area under the curve.

EAK5s ‘s predictive power was compared with CRP’s. We used the inflammation status assessed from clinical data without biological data (to exclude CRP). One hundred and fifty patients had both their clinical inflammation statuses assessed and their CRP levels measured. Areas under the curve (AUC) were 0.85 (0.78–0.91) for EAK5s and 0.81 (0.74–0.88) for CRP. The two curves were significantly different (p < 0.0001), with EAK5s having a better predicting power than CRP (Fig. 1).

Fibrinogen, another mediator of inflammation, was measured in 81 patients. Fibrinogen level was not correlated to EAK5s (r^2^ for linear correlation = 0.35).

### Ultrafast EAK

**Figure 2:**
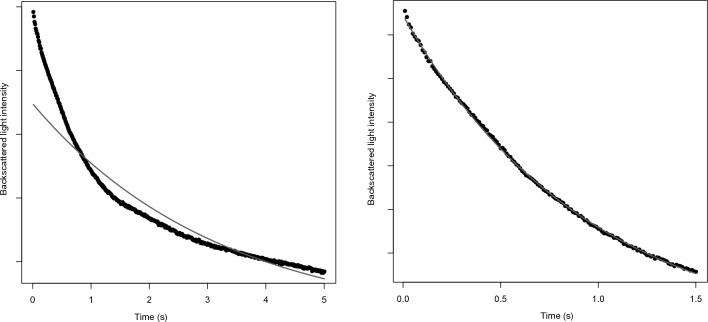
patient with "slow" EAK5s and ultrafast EAK: left panel: the exponential curve (thin curve) fits the 5 s syllectogram (r^2^ = 0.94) poorly. Right panel: near perfect exponential fitting of the 1.5 s syllectogram (r^2^ > 0.99).

For 22 patients the best fit was with the 1.5s model which explained more than 99% of the variance, while the 5s model explained less than 99%. These patients had ultrafast EAK. Mean EAK5s for these patients was 1.53 ± 0.20 s. All these patients’ EAK5s were less than the inflammation threshold except for one patient whose 5 s segment was poorly fitted with the monoexponential model (r^2^ = 94%) with an EAK5s at 2.12 s (Fig. 2). This patient was the only one discharged in this group (diagnosis of gastroenteritis and assessed as with inflammation) and he came back 4 days later with pneumonia eventually treated by antibiotics.

The characteristics of the patients with ultrafast EAK are summarized in Table 1. One patient died of septic shock at day 3, the only patient of the cohort who died during the follow-up period. Nineteen patients were assessed as having inflammation, one as having no inflammation, and three without a clear status.

**Table 1:** Characteristics of the patients with ultrafast EAK.

Total	Mean age (years)	Women	Cancer	Lung inf	Septic shock	Palu	Infect	Pancr	AKF	AHF	CLI	Other
22	67	11	3	6	1	1	2	1	1	2	1	4
		50%	14%	27%	5%	5%	9%	5%	5%	9%	5%	18%

*Lung inf.* infectious pneumopathy, *Palu* neuropaludism, *Infect* other infections, *Pancr* acute pancreatitis, *AKF* acute kidney failure, *AHF* acute heart failure, *CLI* critical leg ischemia.

### EAK test

**Figure 3:**
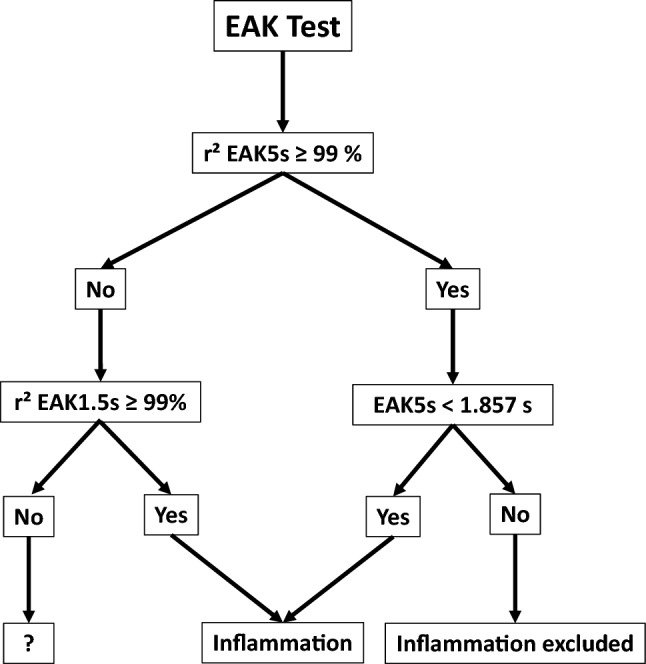
EAK test for inflammation. Inflammation is detected when either EAK5s < 1.857 s (with r ^2^ ≥ 99%) or the EAK is ultrafast (r^2^1.5s ≥ 99%).

If we both only consider the measure of EAK5s when r^2^ is at least of 99% and apply the hypothesis that every patient with ultrafast EAK has inflammation (Fig. 3), the test sensitivity is 73.9%, its specificity is 85.1%, the ppv is 69.9%, and the npv is 87.4%, a slight improvement compared to EAK5s measure alone.

### EAK and infection

Since infection is the leading cause of inflammation in our study, we tested EAK5s’s predicting power for infection. Thirty-five patients had a clinically proven infection (local infection without systemic signs were excluded). Mean EAK5s values were 2.07 ± 0.4 s and 1.74 ± 0.27 s for no infection and infection groups respectively (p < 0.0001). ROC curve AUC was 0.76 (95%CI: 0.68–0.84), with 67.7% (61.6–73.3%) specificity and 75% (60.1–88.9%) sensitivity.

The Youden threshold was 1.880 s, close to the threshold for inflammation. For this threshold, the ppv of EAKs was 25.4% and the npv 94.6%. If we apply the 1.857 s threshold for inflammation, the ppv and npv are 26.3% and 94.3% respectively.

In other words, among patients visiting the ED, an EAK5s greater than 1.86 s excludes a systemic infection with a probability of 94%.

### EAK distribution across diseases

**Figure 4:**
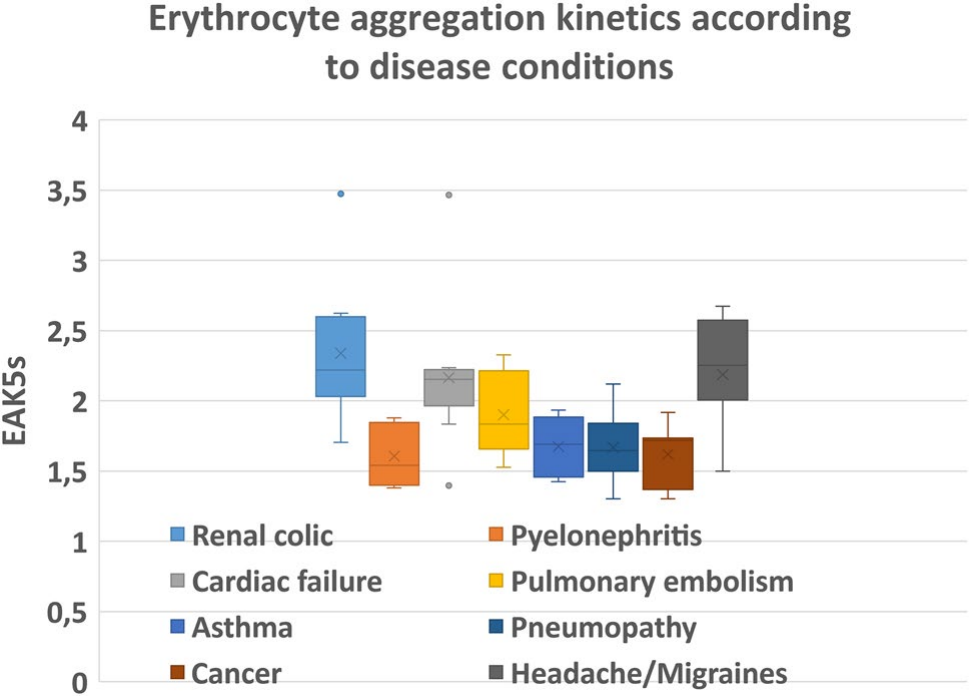
Box-plot of EAK5s in disease groups (71 patients).

Seventy-one patients could be clearly assigned to a disease group of significant size (Fig. 4). Twenty-three patients with acute painful conditions such as renal colic (n = 12), and migraine or common headache (n = 11), had mean EAK5s = 2.27 ± 0.42 s. Thirty-two patients with either pyelonephritis (n = 5), pneumopathy (n = 14), asthma (n = 5) or active cancer (n = 8) had mean EAK5s = 1.77 ± 0.51 s (p < 0.0001 for this two- group comparison). Patients with pulmonary embolism (n = 6) or cardiac failure (n = 10) had a broad range of EAK5s (from 1.53 to 2.29 s and 1.48 to 2.54 s respectively).

## Discussion

Since EA is known to be accelerated with inflammation, the EAK test was used for a broad range of symptoms and disease conditions which could have inflammation in their causal pathways. Therefore, as for any biomarker (for example BNP, troponin, D dimers, CRP etc.), EAK test results should be interpreted while taking into account the clinical context. This is even more important for a systemic pathophysiological phenomenon’s biomarker like inflammation.

### Erythrocyte aggregation and inflammation

**Figure 5:**
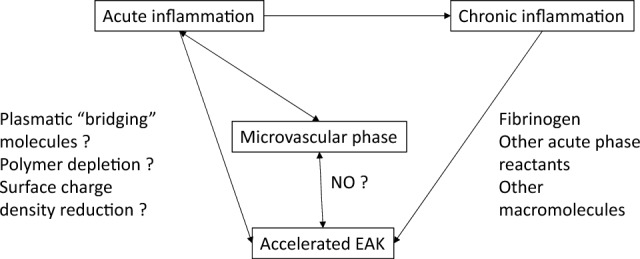
potential mechanisms linking EAK and both acute and chronic inflammation.

EA takes place in low shear stress conditions such as in venous circulation. In addition, an important physiological effect of EA occurs in the arterial microvessels where it leads to a decrease in blood viscosity by increasing the Fahraeus-Lindqvist effect^6^. Previously, we were able to show that the magnitude of EA can modulate the coronary blood flow in a fully vasodilated coronary bed: a physiological degree of EA increases the flow beyond both non- and hyper-aggregation^7^. Therefore, EA impacts the microvascular resistance; EA modifies the distribution of red-blood cells and plasma in the microvascular networks^8,9^, and probably also modifies the flow of leukocytes and their margination^10^. Furthermore, EA can modulate vascular tone by decreasing NO synthesis^5,11^. So, EA, through its hemorheological effects and its action on microcirculation, can directly modulate the inflammation process at its earliest (microvascular) phase. On the contrary, CRP is synthetized by the liver in response to proinflammatory cytokine^12^. Therefore, CRP only begins to increase 6 h after a surgical aggression and needs a few days to peak^13^. This is consistent with the finding that EAK is a better predictor of inflammation than CRP in emergency settings. The link between EA and inflammation is complex. The mechanisms of EA were not fully understood. Fibrinogen appears to play a key role by bridging RBC membranes with a specific mechanism involving GPIIbIIIa receptors^14,15^. However, fibrinogen is unlikely to play a major role in the acceleration of EAK in acute inflammation (emergency setting): fibrinogen’s plasmatic concentration rises long after CRP’s^16^ and fibrinogen level is poorly correlated with EAK in this study. Other unknown “bringing” molecules secreted at the very early phase of the inflammation process could explain the accelerated EAK that we observed. Other mechanisms such as polymer depletion at the erythrocyte surface or surface charge density reduction could be at play^5,17^. Nevertheless, a high fibrinogen level will accelerate EA, so an accelerated EAK test might be expected in long lasting inflammatory conditions. Figure 5 summarizes these mechanisms.

EAK test’s sensitivity and specificity for detecting inflammation are good (better than CRP’s) but not as good as those expected for a “Gold standard” test. Several potential reasons can be listed:

- The inflammation status allocated to the patients by the investigators is not a definitive status.
- Besides the usual limits of any expert-dependent criterium, some underlying inflammatory processes might not be clinically detectable in the short period of observation, especially those associated with cancer or chronic infection.
- Because EA is more likely associated with the initial vascular phase of inflammation, some patients may have early inflammation without any other clinical or biological sign observable during their several-hour visit to the ED.

This bias and the one explained in the paragraph above increase the false-positive rate and, thereby, decrease both the calculated specificity and ppv of the EAK test.

We hypothesize that all patients with an ultrafast EAK have inflammation. This hypothesis seems reasonable since no other known mechanism can explain such a fast EAK.

### Clinical implication

Since EAK is a powerful predictor of inflammation, point-of-care EAK is a useful tool for first-line triage of patients when inflammation is suspected. The high npvs of the EAK test can support the exclusion of inflammation and systemic infection in patients with EAK5s above the threshold.

### Limitation

Only 268 patients were included, so the study lacked power to identify differences even in frequently presenting conditions such as chest pain, dyspnea, or other symptoms. Two investigators assessed each patient’s inflammation status based on the clinical chart; therefore, this assessment was clearly investigator dependent. It should be noted, however, that the investigators were blind to both EAK and CRP test results during the process. Furthermore, the same process was used for EAK and CRP, so the same limitation applies to both.

## Materials and methods

### Clinical study protocol

#### Patient population

Successive adult patients visiting Lariboisiere Hospital’s emergency department during 11 selected days (dayshifts) and needing a blood exam were included.

#### Primary objective

- To describe EAK’s distribution across diagnoses. After analyzing the EAK curves obtained from patients during the first three days, the primary hypothesis was more precisely defined as: EAK5s powerfully predict inflammation.

For each patient:

- The imaging and biological tests performed during the visit to the ED were collected along with the diagnoses established afterward.
- Patients’ conditions were evaluated through both interviews by phone at day 7 and clinical charts when available.
- Two clinical investigators independently and blindly determined each patient’s clinical inflammation status by reviewing their clinical charts (without and with biological data).

The clinical protocol was approved by the appropriate committee according to French law (CPP Ile-de-France VI, Groupe Hospitalier Pitié-Salpêtrière, 4 bâtiment de la Force, 47 boulevard de l’Hôpital 75651 Paris cedex 13) and all subjects gave their informed consent.

#### EAK measurement

The method has been described in detail and validated in a previous publication^18^ and in the Supplementary Information online document. This method is still investigational.

Briefly, blood samples were collected by venipuncture at the point-of-care in a 4ml tube containing EDTA (anticoagulant). This type of regular tube is used in clinical settings for blood cell counts. The tube is inserted in the point-of-care device, which optically measures EAK. The measurement takes 20 s.

We used R software and appropriate functions to fit the data with a mono-exponential model. For each recorded EAK curve (syllectogram), the first 5 s segment was fitted (5s model) and half-life was computed (EAK5s) along with the fitting coefficient r^2^. This coefficient corresponds to the percentage of the variance explained by the model. As inflammation may accelerate EAK to a very high degree, we also fit the first 1.5 s segment and computed the related fitting coefficient (1.5s model). Therefore, we can determine which segment best fits the mono-exponential model. The validating criterium is r^2^ ≥ 99%.

### Statistical methods

For group comparisons we used the Student’s t test. For testing the predictive value of EAK5s we built Receiver Operating Characteristic (ROC) curves and used Delong’s test for computing 95% confidence intervals (95% CI) and comparing ROC curves. We computed decision thresholds using the Youden index (maximalization of the difference between sensitivity and 1-specificity)^19,20^. For all these analyses we used the pROC package for R^20^.

### Statement

All methods were carried out in accordance with relevant guidelines and regulations. All participants gave their informed consents at the inclusion in the study.

### Supplementary Information

Supplementary Information.

## Data Availability

Data from human subjects are managed in accordance with the study protocols and the French Law. All the datasets generated or analyzed during this study are available (request to O. Charansonney).
