# Supplementary material for: Evaluation of a new point-of-care diagnostic test measuring inflammation in emergency settings
Source: Sci Rep. 2023 Nov 9;13:19551. doi: 10.1038/s41598-023-46347-x (PMC10636126; doi:10.1038/s41598-023-46347-x)
Supplement: Supplementary file 1 — Supplementary Information. [file 41598_2023_46347_MOESM1_ESM.docx]

**Supplementary Information**

An in-depth description of the technique is available at: <https://doi.org/10.1038/s41598-022-18410-6> ^1^.

Table 1S summarizes the characteristics of available techniques for measuring erythrocyte aggregation. The EAK test is the only technique which uses a standard 4 ml EDTA tube that is unnecessary to open or sample. The intact blood tube can be used for blood cell counts after EAK measurement. The other EAK test ‘s unique feature is that it measures a kinetics (EAK) and analyzes it with a mechanistic model^1^. Other techniques describe EA kinetics with several parameters such as light intensities, areas under the curve (integrals), and half-lives (descriptive). TEST-1 correlates these parameters with ESR to estimate ESR.

***Table 1S. State of the art for measuring erythrocyte aggregation (EA)***^2–4^. Syllectogram: curve of the evolution of EA in function of time. ERS: Erythrocyte Sedimentation Rate

| Techniques | Description | Measures | Rapidity (results) | Blood sample | Blood handling /transport | Calibration/  Cleaning/ Maintenance | Waste | POC |
| --- | --- | --- | --- | --- | --- | --- | --- | --- |
| EAK Test | Stirring standard unopened tube/Light back-scattering | 5-s-Syllectogram fitting.  Mechanistic model | Fast: 20s | Routine  Re-usable | No | No | No | Yes |
| TEST-1 (automated)  Alifax® | Capillary photometry.  Light transmission | 20-s-syllectogram integral.  Statistical model correlated to ESR | Fast: 20s | Specific, from routine sample (175µl) | Yes, in lab | Yes | Yes | No |
| ESR | Westergren | ESR | Slow: 60 min | Specific | Yes, in lab | Yes | Yes | No |
| Viscosimeters (Myrenne, LORCA) | Blood poured into a viscosimeter. Transmitted or backscattered light | Several indices (light intensity and curve fitting) | Several minutes | Specific | Yes, in research lab  Time consuming | Yes | Yes | No |
| Microchip (RheoScan) | Light transmission | Several indices (light intensity and curve fitting) | Several minutes | Specific | Yes, in lab | Yes | Yes | Non |

EAK test is a point-of-care test.

The characteristics of the research device used in the study are: weight: 1.7 kg, height: 145mm, length: 194mm, width: 150. It can easily fit in an emergency vehicle or on an emergency trolley (Fig. S1).

In this study, the EAK test was performed by the researchers to save ED staff’s time. The test could be performed by nurses after a very short training.


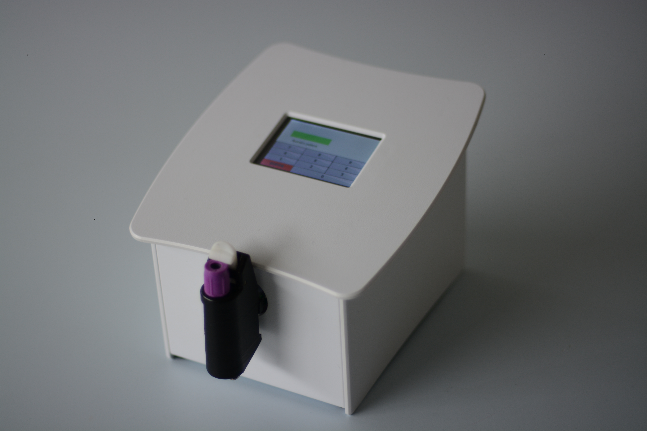


Figure 1S: experimental device

The quality of the measurement was assessed by the fitting coefficient r² which should be >99%.

The EAK test was still investigational in this study. We performed 800 measures on the 275 patients’ blood (only one 4 ml-blood tube per patient). We had a validated measure for 269 patients. Reasons for not having a validated test were: there was no measure for one patient, two patients’ samples were mishandled, and measurement of three patients’ samples failed the quality test. There was no display of the results or r² on the device, so, in the last cases, the operator couldn’t repeat the failed measurements.

**References**

1. Charansonney, O. L., Morel, P., Dufaux, J. & Vicaut, E. Description and validation of a new, simple, easy-to handle, point-of-care technique for measuring erythrocyte aggregation kinetics. *Sci Rep* **12**, 14798 (2022).

2. Hardeman, M. R., Levitus, M., Pelliccia, A. & Bouman, A. A. Test 1 analyser for determination of ESR. 1. Practical evaluation and comparison with the Westergren technique. *Scand J Clin Lab Invest* **70**, 21–25 (2010).

3. Hardeman, M. R., Levitus, M., Pelliccia, A. & Bouman, A. A. Test 1 analyser for determination of ESR. 2. Experimental evaluation and comparison with RBC aggregometry. *Scand J Clin Lab Invest* **70**, 26–32 (2010).

4. Baskurt, Oguz K. *et al.* Comparison of three instruments for measuring red blood cell aggregation. *Clinical Hemorheology and Microcirculation* 283–298 (2009) doi:10.3233/CH-2009-1240.
